# Supplementary material for: Identification of Dirofilaria immitis miRNA using illumina deep sequencing
Source: Vet Res. 2013 Jan 18;44(1):3. doi: 10.1186/1297-9716-44-3 (PMC3598945; doi:10.1186/1297-9716-44-3)
Supplement: Additional file 9 — KEGG pathway of predicted target genes. 9 661 target genes were assigned to 250 KEGG pathways. “Target genes with pathway annotation” represents number and frequency of target genes related to this pathway. “All genes of the species with pathway annotation” represents number and frequency of reference genes related to this pathway. “Pvalue” and “Qvalue” represent P-value before correction and corrected P-value, respectively. [file 1297-9716-44-3-S9.zip › index.htm/Dirofilaria_immites_map/map03060.html]

map03060
